# Social dominance in rats: effects on cocaine self-administration, novelty reactivity and dopamine receptor binding and content in the striatum

**DOI:** 10.1007/s00213-015-4122-8

**Published:** 2015-11-10

**Authors:** Bianca Jupp, Jennifer E. Murray, Emily R. Jordan, Jing Xia, Meg Fluharty, Saurav Shrestha, Trevor W. Robbins, Jeffrey W. Dalley

**Affiliations:** Department of Psychology, University of Cambridge, Downing Street, Cambridge, CB2 3EB UK; Behavioural and Clinical Neurosciences Institute, University of Cambridge, Cambridge, CB2 3EB UK; Molecular Imagine Branch, National Institute of Mental Health, National Institutes of Health, Bethesda, USA; Department of Psychiatry, University of Cambridge, Cambridge, CB2 2QQ UK

**Keywords:** Social status, Psychostimulants, High responder, Anxiety, Impulsivity, Resource competition

## Abstract

**Rationale:**

Studies in human and non-human primates demonstrate that social status is an important determinant of cocaine reinforcement. However, it is unclear whether social rank is associated with other traits that also predispose to addiction and whether social status similarly predicts cocaine self-administration in rats.

**Objectives:**

The objective of this study is to investigate whether social ranking assessed using a resource competition task affects (i) the acquisition, maintenance and reinstatement of cocaine self-administration; (ii) the dopaminergic markers in the striatum; and (iii) the expression of ancillary traits for addiction.

**Methods:**

Social ranking was determined in group-housed rats based upon drinking times during competition for a highly palatable liquid. Rats were then evaluated for cocaine self-administration and cue-induced drug reinstatement or individual levels of impulsivity, anxiety and novelty-induced locomotor activity. Finally, dopamine content, dopamine transporter (DAT) and dopamine D_2_/D_3_ (D_2/3_) receptor binding were measured postmortem in the dorsal and ventral striatum.

**Results:**

Rats deemed socially dominant showed enhanced novelty reactivity but were neither more impulsive nor anxious compared with subordinate rats. Dominant rats additionally maintained higher rates of cocaine self-administration but showed no differences in the acquisition, extinction and reinstatement of this behaviour. D_2/3_ binding was elevated in the nucleus accumbens shell and dorsal striatum of dominant rats when compared to subordinate rats, and was accompanied by elevated DAT and reduced dopamine content in the nucleus accumbens shell.

**Conclusions:**

These findings show that social hierarchy influences the rate of self-administered cocaine but not anxiety or impulsivity in rats. Similar to non-human primates, these effects may be mediated by striatal dopaminergic systems.

**Electronic supplementary material:**

The online version of this article (doi:10.1007/s00213-015-4122-8) contains supplementary material, which is available to authorized users.

## Introduction

Social dominance reflects the tendency of an individual to consistently strive for and achieve desirable outcomes within social encounters (Drews 1993). However, social dominance has also been shown to influence drug reinforcement mechanisms; for example, cocaine intake in humans (Tarter et al. 2007) and other animals (Morgan et al. 2002). Consequently, social status and attendant social stress are viewed as important contributory factors to the initiation, escalation and relapse to drug abuse and addiction (Miczek et al. 2008; Nader et al. 2012; Shaham et al. 2000).

Much previous research has investigated the causal influence of social variables, including social stress, on drug reinforcement mechanisms. For example, social defeat stress in rats enhances the acquisition of cocaine self-administration and increases the motivation to seek and take this drug under a progressive ratio schedule of reinforcement whilst having no effect on ‘dominant’ resident animals (Covington and Miczek 2001, 2005). Consistent with these findings, socially recessive cynomolgus monkeys readily acquire intravenous cocaine self-administration, distinct from dominant monkeys (Morgan et al. 2002). Thus, vulnerability to psychostimulant drugs appears to be a feature of socially subordinate and socially stressed animals. Subordinate, psychostimulant vulnerable monkeys additionally show an increased behavioural reaction to novel objects (Czoty et al. 2010; Riddick et al. 2009), an observation that accords with evidence in rats that high ambulatory activity in a novel environment (the ‘high-responder’ or HR phenotype) predicts an increased propensity to self-administer psychostimulant drugs (Davis et al. 2008; Piazza et al. 1989). However, it is unclear whether social ranking is linked in any way to enhanced novelty-related behaviour in rats. This is a relevant question to ask as cocaine self-administration in HR rats is modulated by social stress (Kabbaj et al. 2001).

To date, the majority of studies investigating the effect of social rank on drug-related behaviours have typically assessed dominance based upon the outcomes of dyadic agonistic interactions, mainly involving measures of aggression in non-human primates (e.g. Kaplan et al. 1982; Miczek 1979; Morgan et al. 2002) or following exposure to social stress (i.e. social defeat stress) in rodents (e.g. Covington and Miczek 2005; Miczek et al. 2011; Yap and Miczek 2007) where dominance hierarchies are experimentally enforced. However, it is not clear from these studies whether the accompanying changes reported in brain neurobiology and drug self-administration simply reflect individual differences in aggression. Whilst aggression is likely to contribute to the development and expression of social dominance (Chase et al. 2002), there is evidence to suggest that these traits may be dissociable (see Francis 1988). In the present study, therefore, we assessed social dominance within group-housed animals based upon individual differences in performance during a resource competition task, specifically for a highly palatable liquid, assigning dominance on the premise that social rank determines priority of access to a desirable resource (Syme 1974). This approach aimed to minimise potential confounds associated with selecting animals purely based upon observed differences in aggressive behaviour and further to permit assessment of individual levels of dominance across the entire social group in which social hierarchy was not experimentally enforced. Following ranking on this task, we then determined whether dominance rank was associated with differences in the acquisition, maintenance and reinstatement of cocaine self-administration and further whether animals selected in this way subsequently exhibited other behavioural traits related to drug self-administration, notably anxiety, novelty seeking and impulsivity (Jupp and Dalley 2014; Lejuez et al. 2008; Weafer et al. 2014). Finally, based on prior neurobiological studies in dominant and subordinate non-human primates (Czoty et al. 2010; Morgan et al. 2002; Nader et al. 2012), we assessed markers of dopamine (DA) neurotransmission in the striatum and frontal cortex as putative neural substrates underlying social dominance in rats.

## Materials and methods

### Experimental subjects

**Fig. 1:**
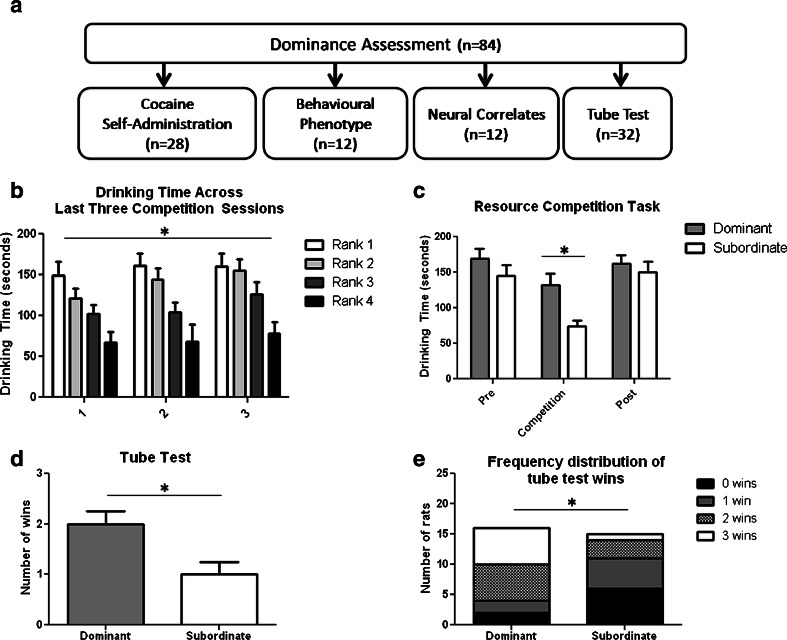
**a** Study design. **b** Drinking times of socially dominant and subordinate rats across the last 3 days of competition *p < 0.05 main effect of rank. **c** Drinking times before, during and after the resource competition task. **d** Tube test performance and **e** frequency distribution of the total number of wins in socially dominant and subordinate rats, *p < 0.05

Eighty-four outbred male Lister Hooded rats (Charles River, Margate, UK), weighing 280–300 g at the beginning of the study, were used. Water was available ad libitum, and sufficient food was provided to maintain body weights at no less than 90 % of free-feeding weights (20 g chow/day). Rats were housed in social groups of four for all experiments, under temperature- and humidity-controlled conditions and a reversed 12-h light/dark cycle (white lights off/red light on at 07:00 hours). All procedures conformed to the UK Animal (Scientific Procedures) Act of 1986 and Council Directive 2010/63EU and were approved by local ethical review at the University of Cambridge. The study design is illustrated in Fig. 1a.

### Resource competition task

Rats were categorised as socially dominant or socially subordinate based on their performance on a resource competition task for a highly palatable liquid, adapted from previous food and water competition studies (Gentsch et al. 1990; Malatynska and Kostowski 1984; Saxton et al. 2011). Following 6–8 weeks of cohabitation to facilitate the development of stable social hierarchies, animals were habituated to the resource competition task during four, once daily, 5-min exposures to the task whereby rats were given solitary access to the performance arena. The arena consisted of a clean empty cage of identical dimensions to the home cage and free access to a drinking bottle with one ballpoint spout containing a highly palatable liquid (Yazoo Strawberry Milkshake, Friesland Campina, Amersfoort, The Netherlands).

Following habituation, rats were challenged with 15 consecutive daily competition sessions, during which time all animals from the same home cage were placed into the performance arena. To minimise fighting during competition sessions, access to the drinking spout was not obstructed and thus, multiple animals could drink at the same time. Animals were individually identified with experimenter-applied tail markings. The final three competition sessions were video-recorded and later scored manually to assess differences in drinking times using ODLog (Macropod Software, Invergowrie, New South Wales, Australia). Dominance ranks were assessed within each group of home-cage rats based on average drinking times across the final three competition sessions. The two animals that drank the most were deemed dominant, whilst the remaining two animals were ranked as subordinate. In subsequent experiments, all animals within an individual social group were enrolled in the same experimental paradigm. In a subset of animals (experiment 3) prior to the first, and following the last day of competition, drinking time was recorded during a session of individual drinking to examine if drinking time during competition was related to differences in an individual’s ability to drink and to assess further whether preference for the highly palatable liquid altered over the duration of the experiment.

### Validation of the resource competition task

Following assessment on the competition resource task, 32 group-housed rats were evaluated for their social rank using a modified version of the social dominance tube task, described previously for use in mice (Lindzey et al. 1961). The tube test assesses dominance in terms of an animal’s choice to advance or retreat following an interaction with a competitor within a tube. Rats were initially habituated to the testing apparatus, which consisted of a 10 cm (diameter) by 30 cm (length) red transparent plastic tube, of sufficient size to allow one but not two rats to move through the tube. Over two consecutive days, rats were allowed to run through the tube on eight occasions, with alternate trials in which the entry and exit ends were switched.

Competition trials involved simultaneously releasing two competing rats into opposite ends of the tube. These trials were performed in a round-robin design within a social group such that each rat met all cage mates on one occasion (i.e. each rat performed three competition trials). The individual rat that was able to travel forwards through the tube to exit the other side ‘won’ and was deemed dominant; the rat that retreated was considered subordinate. Weekly body weights were recorded during this procedure.

### Experiment 1: effect of social dominance on cocaine self-administration, extinction and cue-induced drug seeking

To investigate the relationship between social status and cocaine self-administration, rats previously screened on the resource competition task (*n* = 14 dominant; *n* = 14 subordinate) were trained to self-administer cocaine via the intravenous route. Rats were surgically implanted under ketamine (0.1 ml/100 g body weight; Vet Drug, UK), xylazine (0.05 ml/100 g body weight; Vet Drug) anaesthesia with a chronic indwelling catheter (CamCaths, Cambridge, UK) into their right jugular vein using aseptic technique as described previously (Thomsen and Caine 2005). Rats were weighed and catheters flushed daily with 0.1 ml of 10U heparinised saline (CP Pharmaceuticals Ltd., Wrexham, UK) for the duration of the experiment.

Rats were allowed to recover for 1 week following surgery prior to commencing intravenous drug self-administration. Acquisition of cocaine self-administration was assessed using a two-lever procedure on a fixed ratio-1 (FR-1) schedule of reinforcement over a period of ten daily 2-h sessions conducted in operant chambers (Med Associates Inc., Vermont, USA) controlled by WhiskerServer software (Cardinal and Aitken 2010). Active lever responses were paired with the contingent illumination of a cue light above the active lever for 4 s and resulted in a single cocaine infusion (0.2 mg/kg/infusion) and retraction of both levers for a period of 30 s. Inactive lever responses were recorded but had no programmed effect. Rats were limited to a maximum of 90 cocaine infusions per session.

A subset of rats (*n* = 6 dominant; *n* = 6 subordinate) that had acquired cocaine self-administration (≥50 cocaine infusions, ≥75 % active lever discrimination over the final 3 days of acquisition) were then tested for average FR-1 responding, over three consecutive days, for three doses of cocaine (0.1, 0.2, 0.4 mg/kg/infusion). Dose order was carefully counterbalanced within and between groups using a Latin square design.

Finally, the rats that were tested for cocaine dose responsiveness were exposed to ten consecutive sessions of extinction training during which active lever responding was no longer accompanied by the contingent presentation of the cue light or by an infusion of cocaine. All other parameters remained identical to those used during acquisition. Following extinction (<30 % active lever responding during maintenance over three consecutive days), rats underwent a single cue-induced reinstatement session where active lever responses again resulted in the presentation of the cue light. However, the delivery of cocaine was again withheld.

### Experiment 2: effect of social status on novelty reactivity, anxiety and impulsivity

To assess the relationship of social dominance with other addiction-relevant traits, rats previously screened on the resource competition task (*n* = 6 dominant; *n* = 6 subordinate) were assessed in the following order: (1) locomotor activity in a novel environment (Piazza et al. 1990); (2) trait anxiety in a light-dark shuttle box (Crawley and Goodwin 1980); and (3) impulsivity on the five-choice serial-reaction time task (Robbins 2002). Each behavioural procedure was separated by 1 week during which time rats were maintained in their home cage on a food-restricted diet and free access to water.

### Locomotor reactivity to novelty

Novelty reactivity was assessed during two 120-min sessions conducted over two consecutive days in photocell locomotor tracking boxes (San Diego Instruments, San Diego, CA, USA) under red light conditions. Total horizontal beam breaks during each session were used as an index of locomotor behaviour.

### Light-dark box

Animals were housed in the testing room for 1 h prior to assessment. The testing apparatus consisted of a box divided into two compartments, one black and completely enclosed, the other white and open at the top. The two compartments were coupled by a small open door in the middle. Animals were placed in the middle of the dark chamber and allowed to freely explore the apparatus for 10 min on one occasion. Sessions were video-recorded and the time spent in each of the compartments recorded using ODLog software.

### Five choice serial reaction time task

Rats were trained on the five-choice serial-reaction time task (5-CSRTT) as previously described (Bari et al. 2008). A PC using WhiskerServer software and FiveChoice client (Cardinal and Aitken 2010) controlled the apparatus (Med Associates Inc., USA). Animals were trained to nose poke into one of five apertures following their illumination; correct responses were rewarded with a food pellet, whilst incorrect responses were punished with a 5-s time-out. Daily sessions consisted of 100 discrete trials. Each trial was initiated by the entry of the animal into the food magazine, resulting in the illumination of the house light. Following an inter-trial interval (ITI) of 5 s, a brief light stimulus (0.7 s in duration) was presented in one of the five apertures. A nose poke into the corresponding aperture was rewarded with delivery of one food pellet (45-mg Noyes dustless pellets, Sandown Scientific, Middlesex, UK). Failure of the animal to respond within 5 s (omission) or a nose poke into the incorrect aperture (incorrect response) resulted in a 5-s time-out, during which time no new trials could be initiated and the house light was extinguished. Nose pokes completed prior to onset of the light stimulus (premature or impulsive responses) also resulted in a 5-s time-out. Following acquisition of the task (≥80 % correct responding; ≤20 % omissions), rats were challenged with a fixed, long ITI session of 7 s to increase the frequency of premature responses (Dalley et al. 2007).

### Experiment 3: neural correlates of social dominance

To investigate neurochemical correlates of social dominance and subordination, receptor binding and neurochemical markers of cortico-striatal monoaminergic neurotransmission were measured in a subset of dominant (*n* = 6) and subordinate (*n* = 6) animals using ex vivo autoradiography and high-performance liquid chromatography (HPLC) and electrochemical detection.

Animals were terminally anaesthetised with sodium pentobarbital (1.5 ml, 200 mg/ml i.p.), decapitated and their brains rapidly excised and frozen over liquid nitrogen. All brains were stored at −80 °C until processed. One 150-μm and eight serial 30-μm sections were collected from the following levels relative to bregma (Paxinos and Watson 2007): +3.72 mm (containing prefrontal/orbitofrontal cortical structures); +2.28 mm (containing rostral striatal structures). Cryosections were mounted onto super-frost plus slides (Menzel Glasser, Braunschweig, Germany), three per slide, such that each slide contained sections from three animals in a randomised order. Slides were allowed to dry overnight before being stored at −80 °C.

Bilateral samples of brain tissue (0.4–2 mg) were removed from the thick (150 μm) cryosections using a stainless steel micro-punch (0.75-mm diameter). Regions of interest comprised the nucleus accumbens core (NAc core) and shell (NAc shell), dorsal striatum, infralimbic cortex, prelimbic cortex, orbitofrontal cortex and anterior cingulate cortex. Tissue aliquots were weighed and homogenised in 100 μl of 0.2 M perchloric acid and centrifuged at 6000 rpm for 10 min. An aliquot of the resultant supernatant (25 μl) was injected onto the HPLC system (ESA pump, ESA Coulochem II detector, Gilson 234 autoinjector) as described previously (Dalley et al. 2002) for analysis of DA, dihydroxyphenylacetic acid (DOPAC), noradrenaline (NA), serotonin (5-HT) and 5-hydroxyindoleacetic acid (5-HIAA).

Autoradiographic binding for the dopamine D_2_/D_3_ (D_2/3_) receptor, dopamine transporter (DAT) and serotonin transporter (5-HTT) was conducted using the same conditions as described previously (Jupp et al. 2013). In brief, serial, duplicate sections were incubated with [^3^H]-raclopride (Perkin Elmer, MA, USA) for D_2/3_ binding or [^125^I]-RTI-55 (Perkin Elmer) in the presence of either fluoxetine or nomifensine for DAT and 5-HTT binding, respectively. Sections were dried and opposed to a tritium-sensitive phosphoimaging plate (Fujifilm, Tokyo, Japan) or film (Kodak BioMax MR) together with an appropriate microscale standard ([^3^H], Amersham Biosciences, Freiburg, Germany; [^125^I], American Radiolabeled Chemicals, St. Louis, USA). Autoradiographs were digitised and a region-of-interest analysis conducted using Image J (Abramoff et al. 2004) following calibration with microscale standards. Binding (in mol/mg tissue) for all ligands was assessed bilaterally in the dorsal striatum, NAc core and NAc shell by an operator blind to the experimental group. Specific receptor binding was calculated by subtracting non-specific binding from total binding for each region of interest. Results are presented as average binding for left and right hemispheres.

### Statistical analysis

Behavioural data were primarily analysed using repeated measures analysis of variance (ANOVA: SPSS, version 21, IBM) with ‘rank’ as the between-subjects factor and ‘lever’, ‘dose’ and ‘session’ and ‘side’ as the within-subjects factors. Results of the tube test were analysed using a *t* test. Frequency distribution of the number of wins on the tube test vs dominance rank was analysed using Fisher’s exact test. Receptor binding and neurochemical levels postmortem were assessed by two-way ANOVA (social rank and hemisphere) for each brain region of interest. Significant main effects and interactions were further analysed using ANOVA and post hoc *t* tests corrected for multiple comparisons (Bonferroni). Significant violations from the assumption of sphericity were corrected using the Huynh-Feldt epsilon to allow more conservative comparisons through adjusted degrees of freedom. The alpha criterion value was set at 0.05.

## Results

Drinking times were found to be stable across the last 3 days of competition (Fig. 1b). ANOVA revealed a main effect of rank (*F*_(3,80)_ = 3.473, *p* < 0.0001), but no main effect of time (*F*_(2,80)_ = 3.120, *p* > 0.05) or rank × time interaction (*F*_(6,80)_ = 0.403, *p* > 0.05). Figure 1c shows the drinking times before, during and after competition for a highly palatable liquid for the rats used in experiment 3 assessed as either dominant or subordinate. ANOVA revealed a main effect of session (*F*_(2,22)_ = 21.13, *p* < 0.0001) and a rank × session interaction (*F*_(2,22)_ = 3.23, *p* < 0.05). Drinking times were significantly prolonged in dominant rats compared with subordinate rats during the competition phase (Bonferroni post hoc test *t* = 3.21, *p* < 0.05) but importantly did not differ when rats were tested individually before and after the competition phase. Body weights also did not vary between dominant and subordinate rats during the study (rank *F*_(1,66)_ = 0.28, *p* > 0.05). Secondary assessment of dominance using the social dominance tube task found that rats deemed dominant on the competitive drinking task won a significantly greater number of trials than subordinate rats (*t* = 2.83, *p* < 0.05; Fishers exact test *p* < 0.05) (Fig. 1d, e).

**Fig. 2:**
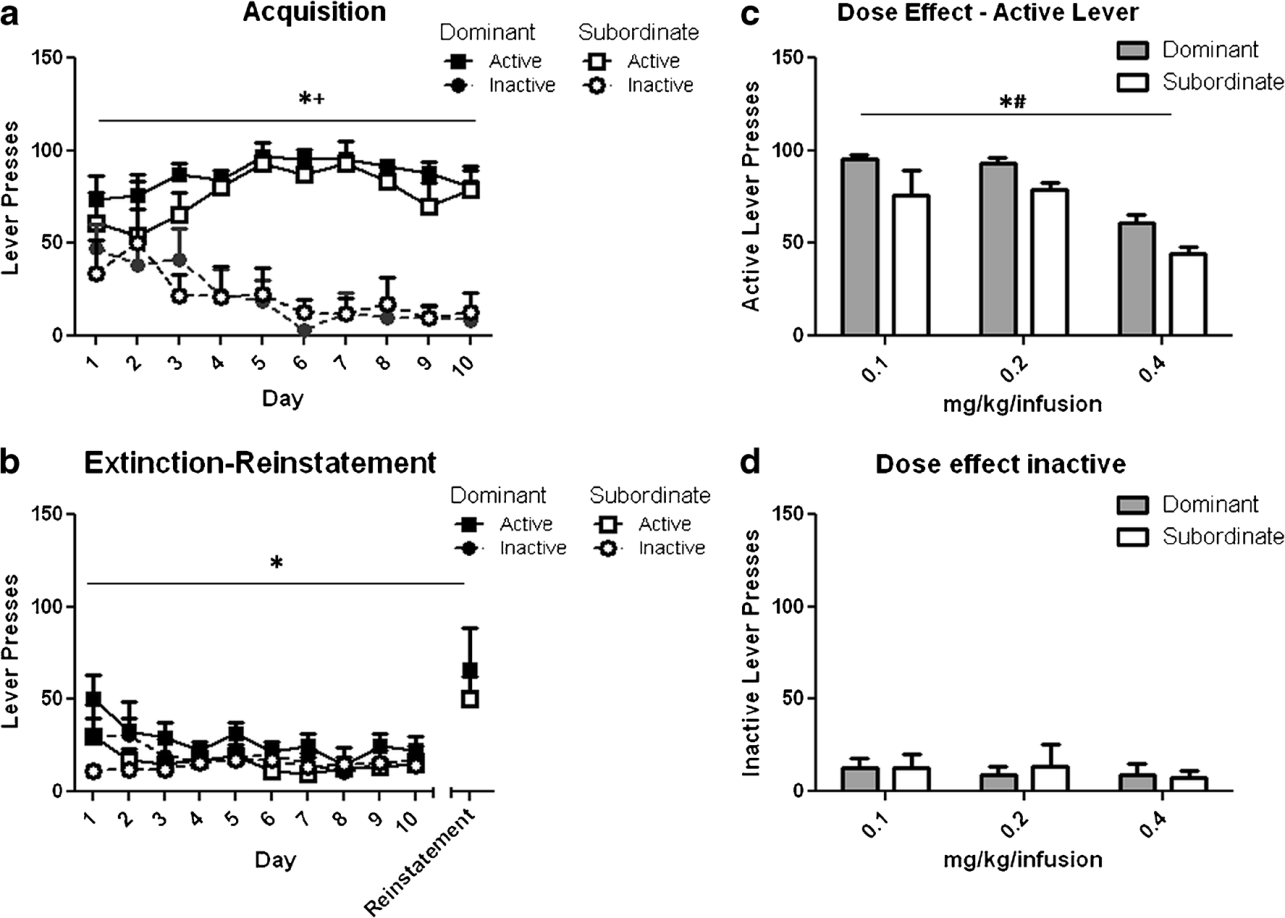
**a** Acquisition of cocaine self-administration *p < 0.05 main effect lever, +*p* < 0.05 lever × day interaction. **b** Extinction and cue-induced reinstatement *p < 0.05 main effect of day. **c** Active lever and **d** inactive lever responses during dose response testing. *p < 0.05 main effect of rank, #*p* < 0.05 main effect of dose, in socially dominant and subordinate rats

All rats acquired intravenous cocaine self-administration (lever *F*_(1,20)_ = 4.053, *p* < 0.001; lever × day interaction *F*_(9,20)_ = 5.02, *p* < 0.0001). However, there was no significant effect of social rank as assayed using the competitive drinking task on the acquisition of this behaviour (rank *F*_(1,20)_ = 0.518, *p* = 0.47; Fig. 2a). Active responding for cocaine was modulated by infusion dose (*F*_(2,20)_ = 26.19, *p* < 0.001) with decreased responding for the highest dose evaluated (0.4 mg/kg/infusion). Dominant rats exhibited significantly increased responding on the active lever compared with subordinate rats (rank *F*_(1,20)_ = 6.57, *p* < 0.05; Fig. 2c), with no significant effects with respect to the inactive lever (Fig. 2d) or the extinction-reinstatement procedure (rank *F*_(1,20)_ = 2.21, *p* = 0.17) (Fig. 2b).

**Fig. 3:**
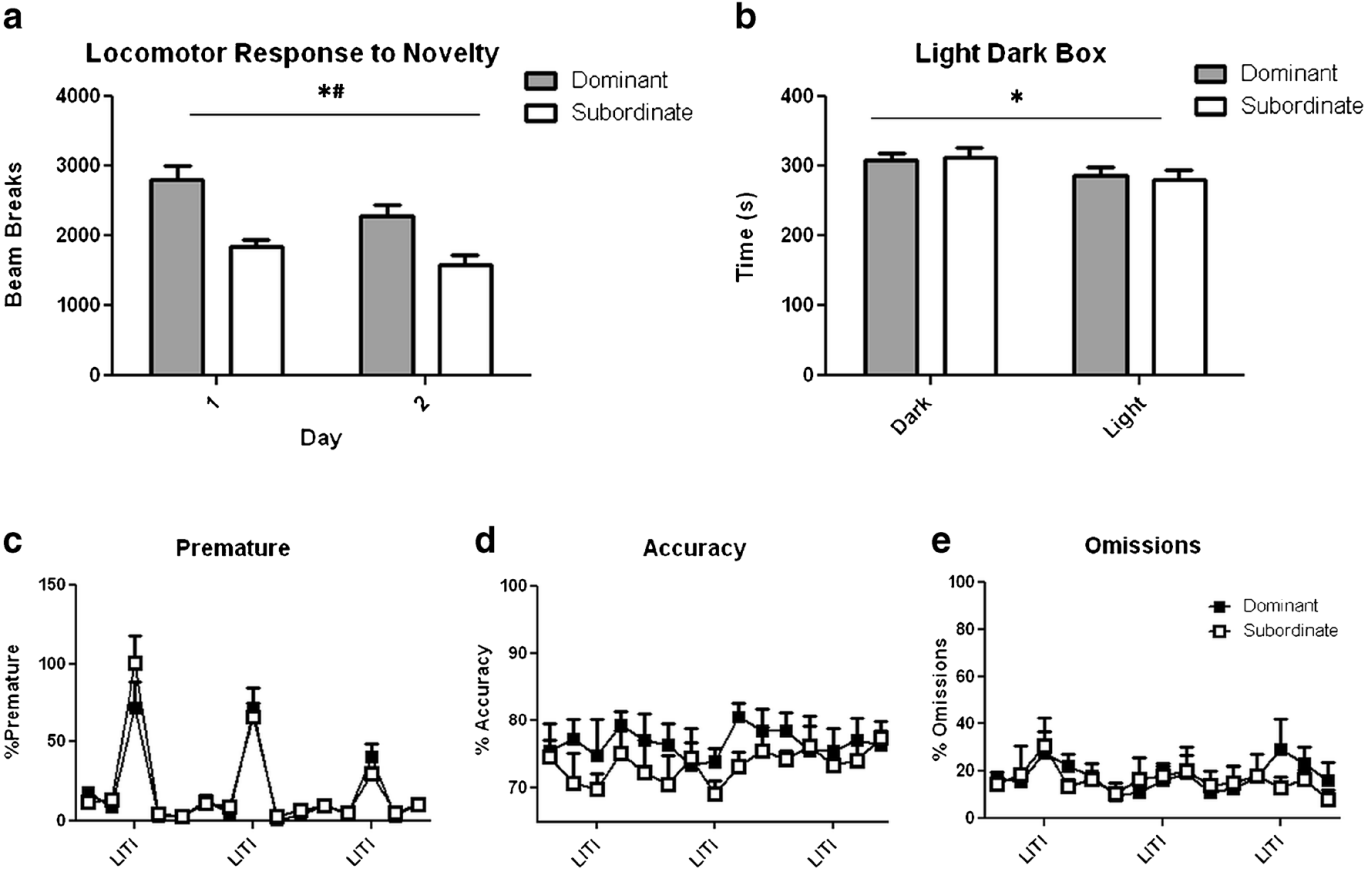
**a** Novelty-induced locomotor activity *p < 0.05 main effect of rank, #*p* < 0.05 main effect of day. **b** Light/dark box performance *p < 0.05 main effect of side and **c** premature responding **d** accuracy and **e** omissions on the five-choice serial-reaction time task in socially dominant and subordinate rats

Rats ranked socially dominant demonstrated an enhanced reaction to novelty, specifically as shown by increased locomotor activity when exposed to a novel environment compared with subordinate rats (rank *F*_(1,20)_ = 6.28, *p* < 0.05; session *F*_(1,20)_ = 19.08, *p* < 0.0001, Fig. 3a). Whilst rats spent a significantly greater proportion of time in the dark side of the dark-light box (side *F*_(1,20)_ = 6.299, *p* < 0.05), no significant difference was observed between dominant and subordinate rats (rank *F*_(1,20)_ = 0.007, *p* = 0.94, Fig. 3b). Furthermore, no significant differences were observed between dominant and subordinate rats on the 5-CSRTT (Fig. 3c–e). As expected, however, there was a main effect of session on premature responding (session *F*_(14,10)_ = 38.64, *p* < 0.0001), reflecting the effect of a long ITI to increase this measure of impulsivity (see Dalley et al. 2007).

**Fig. 4:**
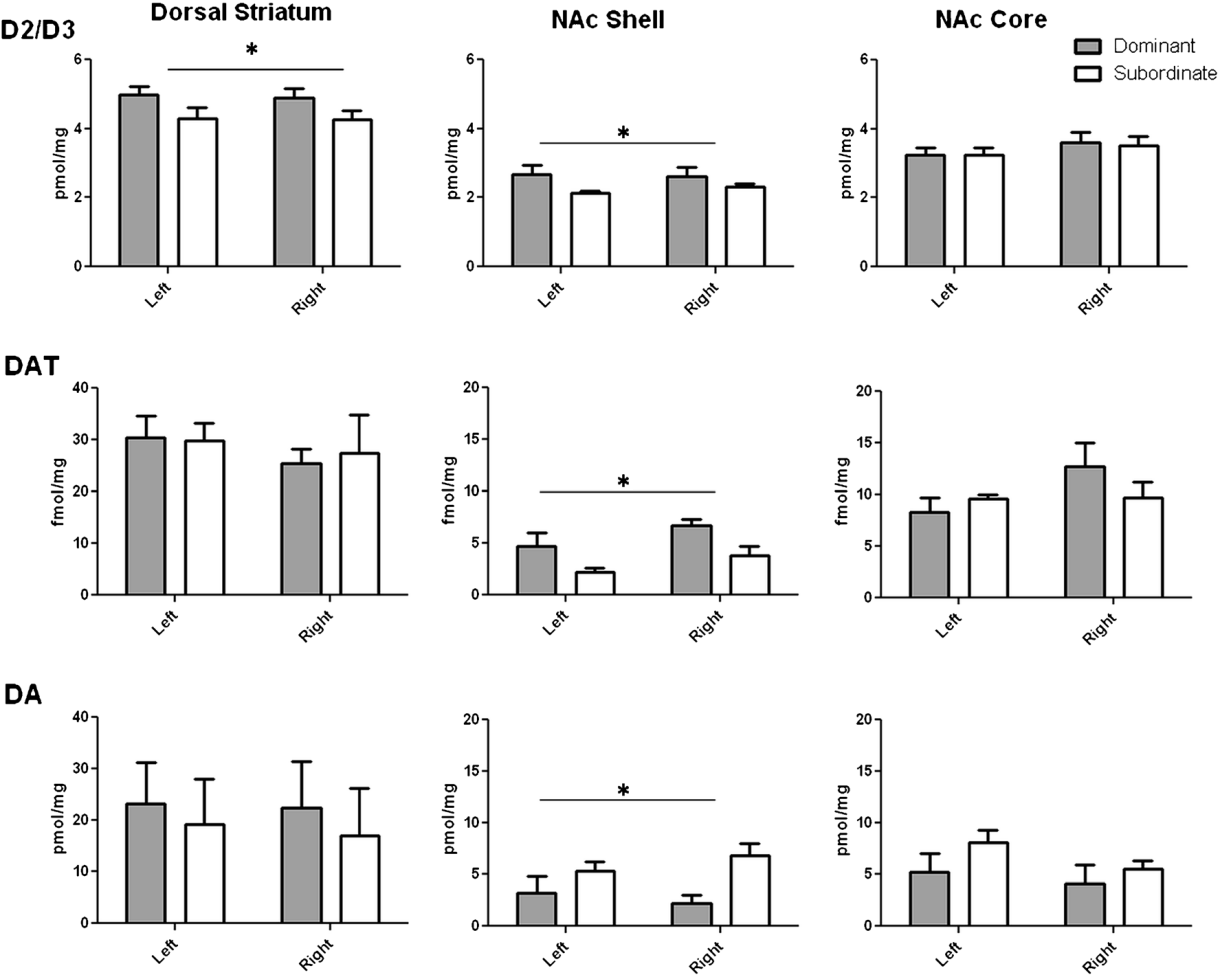
Quantitative assessment of D_2/3_ receptors, DAT and DA content in the dorsal and ventral striatum of socially dominant and subordinate rats, *p < 0.05 main effect of rank

Autoradiographic binding at D_2/3_ receptors, DAT and 5-HTT in the dorsal and ventral striatum are shown in Fig. 4. D_2/3_ receptor binding was significantly greater in the dorsal striatum and NAc shell of dominant rats compared with subordinate rats (dorsal striatum, rank *F*_(1,20)_ = 5.54, *p* < 0.05; NAc shell, rank *F*_(1,20)_ = 5.14, *p* < 0.05). DAT binding was also significantly greater in dominant animals in the NAc shell (rank *F*_(1,20)_ = 6.44, *p* < 0.05). No other significant differences were observed in D_2/3_ receptors, DAT or 5-HTT binding (Supplementary Table 1).

Monoamine levels were also assessed in dominant and subordinate animals bilaterally within the dorsal and ventral striatum and regions of the frontal cortex including anterior cingulate, prelimbic, infralimbic and orbitofrontal cortices. DA levels in the NAc shell were significantly less in dominant than subordinate animals (rank *F*_(1,20)_ = 7.69, *p* < 0.05, Fig. 4). However, no other differences were found with respect to other neurotransmitters and their primary metabolites (Supplementary Tables 2 and 3).

## Discussion

Our findings demonstrate that social dominance in male rats, as assessed using resource competition for a highly palatable liquid, is associated with higher rates of intravenous cocaine self-administration, but predicts neither the acquisition of this operant behaviour nor cue-induced reinstatement of drug-seeking responses following extinction. Rats assessed as socially dominant using this paradigm did however show enhanced novelty-induced locomotion compared with subordinate rats and thus would appear to share features of the HR phenotype shown previously to more readily acquire psychostimulant self-administration (Piazza et al. 1989). Notably, however, the behavioural contrast between the two social groups in the present study did not extend to measures of anxiety or impulsivity and did not predict differential rates of acquisition of cocaine self-administration. Our neurochemical analysis revealed higher D_2/3_ receptor binding in the dorsal striatum of dominant rats together with greater D_2/3_ receptor and DAT binding and diminished and DA content in the NAc shell. These findings support analogous studies in non-human primates showing marked differences between dominant and subordinate animals with respect to cocaine self-administration (Morgan et al. 2002; Nader et al. 2012), novelty reactivity (Czoty et al. 2010) and markers of striatal DA transmission (Czoty et al. 2010; Morgan et al. 2002; Nader et al. 2012). However, as discussed below, the precise effect of social status on cocaine self-administration is complex and may potentially reflect a dissociable relationship between non-human primates and rats.

Positron emission tomography studies have shown that the uptake of the high-affinity D_2/3_ receptor ligand [^18^F]fluoroclebopride is increased in the striatum of socially dominant cynomolgus monkeys compared with subordinate animals (Grant et al. 1998; Morgan et al. 2002). However, when housed individually, D_2/3_ receptor binding was no different between dominant and subordinate animals (Morgan et al. 2002) suggesting that the divergent regulation of striatal D_2/3_ receptors was a consequence of establishing social hierarchies rather than a stable pre-existing variation. The present findings support and extend these earlier studies by showing that socially dominant rats, as assessed on a resource competition task, likewise exhibit elevated D_2/3_ receptor binding in the dorsal striatum and nucleus accumbens (NAcb) shell. The mechanism underlying the observed difference in D_2/3_ receptor binding between social ranks is unclear but may reflect a compensatory consequence of reduced stress and striatal DA release (Shively 1998; Shively et al. 1997) in animals emerging as the dominant member of a social group. The transition to social dominance may thus recruit similar dopaminergic mechanisms in non-human primates and rats.

Nevertheless, our findings reveal two interesting departures from previous studies of social dominance in non-human primates. Firstly, dominant rats in the present study maintained higher rates of intravenous cocaine self-administration than subordinate rats, a finding that stands in marked contrast to non-human primates where cocaine more effectively supported cocaine self-administration in subordinate monkeys than dominant monkeys, with a leftward shift in the cocaine dose-response curve (Morgan et al. 2002) and studies in socially defeated rats demonstrating escalated cocaine use (Covington and Miczek 2005). Secondly, subordinate monkeys have been shown more rapidly to explore novel objects than dominant monkeys (Czoty et al. 2010; Riddick et al. 2009), perhaps consistent with the observation that high locomotor activity in singly housed monkeys predicts later social subordination (Morgan et al. 2000). Our finding of increased novelty-induced locomotion in rats dominant on the competition task thus appears to conflict with these earlier reports in non-human primates. Explanations for this apparent discrepancy, aside from obvious species differences, include the disparity in the method used to assess social dominance in previous studies in non-human primates when compared to the current approach in rats, with the former based mainly on aggressive dyadic encounters, which may co-select for differing traits than the present incentive-based competition procedure. Indeed, individual measures of aggression and outcomes from resource competition are not always correlated (see Syme 1974), suggesting these measures may relate to different modalities of social dominance which may in turn predict divergent aspects of cocaine reinforcement. Importantly for the use of drinking time during a resource competition task as a marker for social dominance, rats assessed as socially dominant using this approach were also found to be dominant on a complementary assay for dominance, the tube test. Further, pre- and post-competition solitary drinking times did not differ between dominant and subordinate rats suggesting that differences during competition were not simply related to individual differences in preference for the palatable liquid or competence in drinking despite potential issues in matching drinking times across different groups of animals.

The establishment of a social hierarchy itself may be an important determinant of individual variation in novelty-induced locomotion. Thus, similar to the modification in D_2/3_ receptor regulation proposed above, the development and expression of social hierarchies and the social stress and coping mechanisms related to this may play a pivotal role in shaping the expression of behavioural traits in group-housed animals. Thus, the formation of a stable social structure may have an important bearing on the expression of novelty seeking and therefore the acquisition and maintenance of cocaine self-administration.

A number of traits have been shown to modulate intravenous cocaine self-administration including impulsivity (Dalley et al. 2007), anxiety (Dilleen et al. 2012; Shively et al. 1997) and novelty seeking and preference (Belin et al. 2011; Czoty et al. 2010; Piazza et al. 2000). In the present study, rats judged to be socially dominant were neither anxious nor impulsive but showed increased novelty-induced locomotion, which may explain their increased rates of cocaine self-administration, particularly as novelty-seeking HR rats that resist social defeat stress also self-administer more cocaine (Kabbaj et al. 2001). Interestingly, following social defeat stress, the acquisition of cocaine self-administration was delayed in HR rats (Kabbaj et al. 2001) demonstrating that even within this behavioural category, individual variation to stress can be observed. Nevertheless, as a group, HR rats are generally less anxious than LR rats (Stead et al. 2006) and whilst they also show reduced delayed discounting impulsivity, are more impulsive on a differential reinforcement of low rates of responding task (Flagel et al. 2010). Collectively, these findings indicate that socially dominant rats, assessed by the resource competition assay, are distinct from the HR phenotype, despite some overlap in novelty-induced behavioural responses.

In addition to perturbations in D_2/3_ receptor binding in the striatum, social dominance in rats was accompanied by increased DAT binding and by reduced DA content in the NAcb shell compared with subordinate animals. These findings are notable for two reasons. Firstly, the reported differences in DAT binding between dominant and subordinate rats are broadly consistent with findings of diminished DAT binding in subordinate group-housed cynomolgus monkeys (Nader et al. 2012). This study however used female monkeys, making direct comparisons with the present study difficult, especially as dominant female monkeys acquired cocaine self-administration at significantly lower doses than subordinate monkeys. Similar to findings in dominant males (Morgan et al. 2002), and the current study, D_2/3_ receptor binding was higher in the striatum of dominant female monkeys compared with subordinate animals. These findings suggest, therefore, that regardless of sex and species, the formation of social hierarchies has broadly similar effects on DAT and D_2/3_ receptors in the striatum. Secondly, intravenous cocaine self-administration in non-human primates has been shown to result initially in reduced DAT binding in the striatum but after a longer exposure, to increased DAT binding in the ventral striatum, particularly the NAcb shell (Letchworth et al. 2001). How this phenomenon interacts with *baseline* differences in DAT binding between dominant and subordinate rats is unknown but as the primary molecular target for cocaine, may contribute to the differing rates of cocaine self-administration between the two groups. The associated reduction in DA content in the NAcb shell perhaps reflects reduced DA transmission in this region leading in turn to a compensatory increase in postsynaptic D_2/3_ receptors.

In humans, psychostimulant addiction is widely associated with reduced D_2/3_ receptor binding and DA release broadly throughout the striatum (Martinez et al. 2004, 2007; Nutt et al. 2015; Volkow et al. 1997). More localised reductions in D_2/3_ receptor binding are present in the NAc of impulsive rats predisposed to compulsive cocaine self-administration (Belin et al. 2008; Dalley et al. 2007) and HR rats (Hooks et al. 1994) susceptible to the acquisition of stimulant self-administration (Piazza et al. 1989). Together, these findings demonstrate that D_2/3_ receptor regulation in the striatum is modifiable by antecedent traits, by social environment and by chronic drug intake. However, our findings of higher D_2/3_ receptor binding in the striatum of dominant rats, which maintained higher rates of cocaine self-administration, present a major challenge in understanding how apparently opponent changes in this receptor subtype causally relate to cocaine reinforcement and dependence. This distinction may have its basis in the divergent regulation of presynaptic and postsynaptic D_2/3_ receptors in the striatum, which may also co-segregate with novelty-seeking and impulsivity traits (Buckholtz et al. 2010; Zald et al. 2008), but further research would be needed to examine this hypothesis.

In conclusion, the present study demonstrates that social dominance in rats, as assessed using a resource competition procedure, is accompanied by marked changes in the striatal dopaminergic systems. Our findings reveal a remarkable correspondence with analogous studies in non-human primates (Morgan et al. 2002; Nader et al. 2012) thereby confirming and extending the conclusion that social status is an important variable underlying individual variation in striatal D_2/3_ receptors. Nevertheless, our findings highlight complexities in the precise influence of social status on intravenous cocaine self-administration that appear to differ between non-human primates and rats. Using dominance rankings based upon priority to access a palatable liquid resource, we show that social dominance co-segregates with increased novelty-induced locomotion, akin to the HR phenotype, and predicts higher rates of cocaine self-administration compared with subordinate rats. However, unlike HR rats, social dominance had no effect on the acquisition of cocaine self-administration and was independent of anxiety and impulsivity. Thus, social dominance in rats, as assessed using a novel resource competition procedure, appears to be a distinct phenotype that may have relevance to the aetiology of psychostimulant addiction.

## Electronic supplementary material

Supplementary Table 1 (DOC 32 kb)

Supplementary Table 2 (DOC 38 kb)

Supplementary Table 3 (DOC 35 kb)

## Abbreviations

5-HTT: Serotonin transporter
DA: Dopamine
DAT: Dopamine transporter
D_2/3_: Dopamine D_2_/D_3_
ITI: Inter-trial interval
NAcb: Nucleus accumbens
HPLC: High-performance liquid chromatography
DOPAC: Dihydroxyphenylacetic acid
NA: Noradrenaline
5-HT: Serotonin
5-HIAA: 5-Hydroxyindoleacetic acid
FR-1: Fixed ratio-1
HR: High responder

## References

[CR1] Abramoff MD, Magalhaes PJ, Ram SJ (2004). Image processing with image J. Biophoton Int.

[CR2] Bari A, Dalley JW, Robbins TW (2008). The application of the 5-choice serial reaction time task for the assessment of visual attentional processes and impulse control in rats. Nat Protoc.

[CR3] Belin D, Mar AC, Dalley JW, Robbins TW, Everitt BJ (2008). High impulsivity predicts the switch to compulsive cocaine-taking. Science.

[CR4] Belin D, Berson N, Balado E, Piazza PV, Deroche-Gamonet V (2011). High-novelty-preference rats are predisposed to compulsive cocaine self-administration. Neuropsychopharmacology.

[CR5] Buckholtz JW, Treadway MT, Cowan RL, Woodward ND, Li R, Ansari MS, Baldwin RM, Schwartzman AN, Shelby ES, Smith CE, Kessler RM, Zald DH (2010). Dopaminergic network differences in human impulsivity. Science.

[CR6] Cardinal RN, Aitken MR (2010). Whisker: a client–server high-performance multimedia research control system. Behav Res Methods.

[CR7] Chase ID, Tovey C, Spangler-Martin D, Manfredonia M (2002). Individual differences versus social dynamics in the formation of animal dominance hierarchies. Proc Natl Acad Sci U S A.

[CR8] Covington HE, Miczek KA (2001). Repeated social-defeat stress, cocaine or morphine. Effects on behavioral sensitization and intravenous cocaine self-administration “binges”. Psychopharmacology (Berlin).

[CR9] Covington HE, Miczek KA (2005). Intense cocaine self-administration after episodic social defeat stress, but not after aggressive behavior: dissociation from corticosterone activation. Psychopharmacology (Berlin).

[CR10] Crawley J, Goodwin FK (1980). Preliminary report of a simple animal behavior model for the anxiolytic effects of benzodiazepines. Pharmacol Biochem Behav.

[CR11] Czoty PW, Gage HD, Nader MA (2010). Differences in D2 dopamine receptor availability and reaction to novelty in socially housed male monkeys during abstinence from cocaine. Psychopharmacology (Berlin).

[CR12] Dalley JW, Theobald DE, Pereira EA, Li PM, Robbins TW (2002) Specific abnormalities in serotonin release in the prefrontal cortex of isolation-reared rats measured during behavioural performance of a task assessing visuospatial attention and impulsivity. Psychopharmacology (Berl) 164:329–340 10.1007/s00213-002-1215-y 12424557

[CR13] Dalley JW, Fryer TD, Brichard L, Robinson ES, Theobald DE, Laane K, Pena Y, Murphy ER, Shah Y, Probst K, Abakumova I, Aigbirhio FI, Richards HK, Hong Y, Baron JC, Everitt BJ, Robbins TW (2007). Nucleus accumbens D2/3 receptors predict trait impulsivity and cocaine reinforcement. Science.

[CR14] Davis BA, Clinton SM, Akil H, Becker JB (2008). The effects of novelty-seeking phenotypes and sex differences on acquisition of cocaine self-administration in selectively bred high-responder and low-responder rats. Pharmacol Biochem Behav.

[CR15] Dilleen R, Pelloux Y, Mar AC, Molander A, Robbins TW, Everitt BJ, Dalley JW, Belin D (2012). High anxiety is a predisposing endophenotype for loss of control over cocaine, but not heroin, self-administration in rats. Psychopharmacology (Berlin).

[CR16] Drews C (1993). The concept and definition of dominance in animal behaviour. Behaviour.

[CR17] Flagel SB, Robinson TE, Clark JJ, Clinton SM, Watson SJ, Seeman P, Phillips PE, Akil H (2010). An animal model of genetic vulnerability to behavioral disinhibition and responsiveness to reward-related cues: implications for addiction. Neuropsychopharmacology.

[CR18] Francis RC (1988). On the relationship between aggression and social dominance. Ethology.

[CR19] Gentsch C, Lichtsteiner M, Feer H (1990). Competition for sucrose-pellets in triads of male Wistar rats: effects of acute and subchronic chlordiazepoxide. Psychopharmacology (Berlin).

[CR20] Grant KA, Shively CA, Nader MA, Ehrenkaufer RL, Line SW, Morton TE, Gage HD, Mach RH (1998). Effect of social status on striatal dopamine D2 receptor binding characteristics in cynomolgus monkeys assessed with positron emission tomography. Synapse.

[CR21] Hooks MS, Juncos JL, Justice JB, Meiergerd SM, Povlock SL, Schenk JO, Kalivas PW (1994). Individual locomotor response to novelty predicts selective alterations in D1 and D2 receptors and mRNAs. J Neurosci.

[CR22] Jupp B, Dalley JW (2014). Behavioral endophenotypes of drug addiction: etiological insights from neuroimaging studies. Neuropharmacology.

[CR23] Jupp B, Caprioli D, Saigal N, Reverte I, Shrestha S, Cumming P, Everitt BJ, Robbins TW, Dalley JW (2013). Dopaminergic and GABA-ergic markers of impulsivity in rats: evidence for anatomical localisation in ventral striatum and prefrontal cortex. Eur J Neurosci.

[CR24] Kabbaj M, Norton CS, Kollack-Walker S, Watson SJ, Robinson TE, Akil H (2001). Social defeat alters the acquisition of cocaine self-administration in rats: role of individual differences in cocaine-taking behavior. Psychopharmacology (Berlin).

[CR25] Kaplan JR, Manuck SB, Clarkson TB, Lusso FM, Taub DM (1982). Social status, environment, and atherosclerosis in cynomolgus monkeys. Arteriosclerosis.

[CR26] Lejuez CW, Zvolensky MJ, Daughters SB, Bornovalova MA, Paulson A, Tull MT, Ettinger K, Otto MW (2008). Anxiety sensitivity: a unique predictor of dropout among inner-city heroin and crack/cocaine users in residential substance use treatment. Behav Res Ther.

[CR27] Letchworth SR, Nader MA, Smith HR, Friedman DP, Porrino LJ (2001). Progression of changes in dopamine transporter binding site density as a result of cocaine self-administration in rhesus monkeys. J Neurosci.

[CR28] Lindzey G, Winston H, Manosevitz M (1961). Social dominance in inbred mouse strains. Nature.

[CR29] Malatynska E, Kostowski W (1984). The effect of antidepressant drugs on dominance behavior in rats competing for food. Pol J Pharmacol Pharm.

[CR30] Martinez D, Broft A, Foltin RW, Slifstein M, Hwang DR, Huang Y, Perez A, Frankle WG, Cooper T, Kleber HD, Fischman MW, Laruelle M (2004). Cocaine dependence and d2 receptor availability in the functional subdivisions of the striatum: relationship with cocaine-seeking behavior. Neuropsychopharmacology.

[CR31] Martinez D, Narendran R, Foltin RW, Slifstein M, Hwang DR, Broft A, Huang Y, Cooper TB, Fischman MW, Kleber HD, Laruelle M (2007). Amphetamine-induced dopamine release: markedly blunted in cocaine dependence and predictive of the choice to self-administer cocaine. Am J Psychiatry.

[CR32] Miczek KA (1979). A new test for aggression in rats without aversive stimulation: differential effects of d-amphetamine and cocaine. Psychopharmacology (Berlin).

[CR33] Miczek KA, Yap JJ, Covington HE (2008). Social stress, therapeutics and drug abuse: preclinical models of escalated and depressed intake. Pharmacol Ther.

[CR34] Miczek KA, Nikulina EM, Shimamoto A, Covington HE (2011). Escalated or suppressed cocaine reward, tegmental BDNF, and accumbal dopamine caused by episodic versus continuous social stress in rats. J Neurosci.

[CR35] Morgan D, Grant KA, Prioleau OA, Nader SH, Kaplan JR, Nader MA (2000). Predictors of social status in cynomolgus monkeys (Macaca fascicularis) after group formation. Am J Primatol.

[CR36] Morgan D, Grant KA, Gage HD, Mach RH, Kaplan JR, Prioleau O, Nader SH, Buchheimer N, Ehrenkaufer RL, Nader MA (2002). Social dominance in monkeys: dopamine D2 receptors and cocaine self-administration. Nat Neurosci.

[CR37] Nader MA, Nader SH, Czoty PW, Riddick NV, Gage HD, Gould RW, Blaylock BL, Kaplan JR, Garg PK, Davies HM, Morton D, Garg S, Reboussin BA (2012). Social dominance in female monkeys: dopamine receptor function and cocaine reinforcement. Biol Psychiatry.

[CR38] Nutt DJ, Lingford-Hughes A, Erritzoe D, Stokes PR (2015). The dopamine theory of addiction: 40 years of highs and lows. Nat Rev Neurosci.

[CR39] Paxinos G, Watson C (2007). The rat brain in stereotaxic coordinates.

[CR40] Piazza PV, Deminiere JM, Le Moal M, Simon H (1989). Factors that predict individual vulnerability to amphetamine self-administration. Science.

[CR41] Piazza PV, Deminiere JM, Maccari S, Mormede P, Le Moal M, Simon H (1990). Individual reactivity to novelty predicts probability of amphetamine self-administration. Behav Pharmacol.

[CR42] Piazza PV, Deroche-Gamonent V, Rouge-Pont F, Le Moal M (2000). Vertical shifts in self-administration dose–response functions predict a drug-vulnerable phenotype predisposed to addiction. J Neurosci.

[CR43] Riddick NV, Czoty PW, Gage HD, Kaplan JR, Nader SH, Icenhower M, Pierre PJ, Bennett A, Garg PK, Garg S, Nader MA (2009). Behavioral and neurobiological characteristics influencing social hierarchy formation in female cynomolgus monkeys. Neuroscience.

[CR44] Robbins TW (2002). The 5-choice serial reaction time task: behavioural pharmacology and functional neurochemistry. Psychopharmacology (Berlin).

[CR45] Saxton KB, John-Henderson N, Reid MW, Francis DD (2011). The social environment and IL-6 in rats and humans. Brain Behav Immun.

[CR46] Shaham Y, Erb S, Stewart J (2000). Stress-induced relapse to heroin and cocaine seeking in rats: a review. Brain Res Brain Res Rev.

[CR47] Shively CA (1998). Social subordination stress, behavior, and central monoaminergic function in female cynomolgus monkeys. Biol Psychiatry.

[CR48] Shively CA, Grant KA, Ehrenkaufer RL, Mach RH, Nader MA (1997). Social stress, depression, and brain dopamine in female cynomolgus monkeys. Ann N Y Acad Sci.

[CR49] Stead JD, Clinton S, Neal C, Schneider J, Jama A, Miller S, Vazquez DM, Watson SJ, Akil H (2006). Selective breeding for divergence in novelty-seeking traits: heritability and enrichment in spontaneous anxiety-related behaviors. Behav Genet.

[CR50] Syme GJ (1974). Competitive orders as measures of social dominance. Anim Behav.

[CR51] Tarter RE, Kirisci L, Kirillova GP, Gavaler J, Giancola P, Vanyukov MM (2007). Social dominance mediates the association of testosterone and neurobehavioral disinhibition with risk for substance use disorder. Psychol Addict Behav.

[CR52] Thomsen M, Caine SB (2005) Chronic intravenous drug self-administration in rats and mice. Curr Protoc Neurosci Chapter 9: Unit 9 20. 10.1002/0471142301.ns0920s32 18428629

[CR53] Volkow ND, Wang GJ, Fowler JS, Logan J, Gatley SJ, Hitzemann R, Chen AD, Dewey SL, Pappas N (1997). Decreased Striatal dopaminergic responsiveness in detoxified cocaine-dependent subjects. Nature.

[CR54] Weafer J, Mitchell SH, de Wit H (2014). Recent translational findings on impulsivity in relation to drug abuse. Curr Addict Rep.

[CR55] Yap JJ, Miczek KA (2007). Social defeat stress, sensitization, and intravenous cocaine self-administration in mice. Psychopharmacology (Berlin).

[CR56] Zald DH, Cowan RL, Riccardi P, Baldwin RM, Ansari MS, Li R, Shelby ES, Smith CE, McHugo M, Kessler RM (2008). Midbrain dopamine receptor availability is inversely associated with novelty-seeking traits in humans. J Neurosci.

