# Supplementary material for: Social dominance in rats: effects on cocaine self-administration, novelty reactivity and dopamine receptor binding and content in the striatum
Source: Psychopharmacology (Berl). 2015 Nov 10;233:579–89. doi: 10.1007/s00213-015-4122-8 (PMC4726718; doi:10.1007/s00213-015-4122-8)
Supplement: Supplementary file 1 — (DOC 32 kb) [file 213_2015_4122_MOESM1_ESM.doc]

**Supplementary Table 1:** Quantitative analysis of D_2/3_ receptor binding, DAT and 5-HTT in dominant and subordinate rats. Binding levels are expressed in fmol/mg of tissue. DS, dorsal striatum; NAc, nucleus accumbens; ACg, anterior cingulate cortex; PrL, prelimbic cortex; IL, infralimbic cortex; OFC, orbitofrontal cortex; NAcbC, nucleus accumbens core; NAcbS, nucleus accumbens shell; HC, hippocampus; BLA, basolateral amygdala. Significant differences (p<0.05) are shown in bold.

| Region | Side | D_2/3_ | | DAT | | 5-HTT | |
| --- | --- | --- | --- | --- | --- | --- | --- |
|  |  | Dom | Sub | Dom | Sub | Dom | Sub |
| DS | Right | **4.91 ± 0.25** | **4.26 ± 0.29** | 25.49 ± 2.78 | 27.44 ± 7.48 | 13.11 ± 6.52 | 14.50 ± 3.22 |
|  | Left | **4.98 ± 0.24** | **4.30 ± 0.41** | 30.46 ± 4.18 | 29.89 ± 3.37 | 13.70 ± 6.46 | 11.48 ± 1.85 |
| NAc Core | Right | 3.92 ± 0.31 | 3.10 ± 0.30 | 10.35 ± 0.40 | 9.72 ± 1.45 | 8.40 ± 2.63 | 13.69 ± 1.95 |
|  | Left | 3.90 ± 0.24 | 3.04 ± 0.03 | 8.27 ± 1.33 | 9.65 ± 0.50 | 12.10 ± 4.20 | 10.43 ± 0.93 |
| NAc Shell | Right | **2.46 ± 0.27** | **2.31 ± 0.09** | **6.70 ± 0.66** | **2.25 ± 0.18** | 22.47 ± 6.98 | 22.94 ± 4.32 |
|  | Left | **2.47 ± 0.36** | **2.12 ± 0.08** | **4.76 ± 1.41** | **3.90 ± 0.86** | 26.14 ± 9.84 | 23.88 ± 3.87 |
