# Supplementary material for: Social dominance in rats: effects on cocaine self-administration, novelty reactivity and dopamine receptor binding and content in the striatum
Source: Psychopharmacology (Berl). 2015 Nov 10;233:579–89. doi: 10.1007/s00213-015-4122-8 (PMC4726718; doi:10.1007/s00213-015-4122-8)
Supplement: Supplementary file 2 — (DOC 38 kb) [file 213_2015_4122_MOESM2_ESM.doc]

**Supplementary Table 2:** Post-mortem levels of NA, DA, DOPAC, 5-HT and 5-HIIA within fronto-cortical regions of dominant (dom) and subordinate (sub) rats. Data are expressed as pmol/mg of tissue. PrL, prelimbic cortex; IL, infralimbic cortex; ACg, anterior cingulate cortex; OFC, orbitofrontal cortex.

| Region | Side | NA | | DA | | DOPAC | | 5-HT | | 5-HIAA | |
| --- | --- | --- | --- | --- | --- | --- | --- | --- | --- | --- | --- |
|  |  | Dom | Sub | Dom | Sub | Dom | Sub | Dom | Sub | Dom | Sub |
| PrL | Right | 1.22 ± 0.26 | 1.38 ± 0.34 | 0.24 ± 0.12 | 0.15 ± 0.04 | 2.17 ± 0.61 | 1.83 ± 0.36 | 0.16 ± 0.04 | 0.20 ± 0.05 | 1.81 ± 0.29 | 1.93 ± 0.32 |
|  | Left | 1.49 ± 0.23 | 1.21 ± 0.10 | 0.19 ± 0.05 | 0.14 ± 0.04 | 2.12 ± 0.21 | 1.77 ± 0.09 | 0.16 ± 0.04 | 0.12 ± 0.02 | 2.58 ± 0.48 | 2.22 ± 0.53 |
| ILC | Right | 1.33 ± 0.39 | 1.56 ± 0.74 | 0.27 ± 0.15 | 0.24 ± 0.11 | 3.54  ± 1.84 | 3.39 ± 1.58 | 0.16 ± 0.03 | 0.30 ± 0.15 | 1.83 ± 0.23 | 2.23 ± 0.50 |
|  | Left | 3.82 ± 1.69 | 1.89 ± 0.41 | 0.40 ± 0.22 | 0.45 ± 0.19 | 4.76 ± 3.04 | 5.51 ± 2.13 | 0.19 ± 0.03 | 0.48 ± 0.24 | 1.78 ± 0.19 | 3.20 ± 0.79 |
| ACg | Right | 1.20 ± 0.32 | 0.87 ± 0.18 | 0.09 ± 0.02 | 0.16 ± 0.10 | 0.87 ± 0.09 | 2.06 ± 0.72 | 0.15 ± 0.03 | 0.09 ± 0.03 | 1.48 ± 0.32 | 1.11 ± 0.28 |
|  | Left | 1.57 ± 0.27 | 1.13 ± 0.20 | 0.25 ± 0.13 | 0.57 ± 0.44 | 3.36 ± 1.26 | 4.88 ± 3.50 | 0.21 ± 0.06 | 0.19 ± 0.05 | 1.81 ± 0.26 | 1.85  ± 0.43 |
| OFC | Right | 1.28 ± 0.47 | 1.24 ± 0.22 | 0.13 ± 0.06 | 0.04 ± 0.02 | 0.54 ± 0.14 | 0.30 ± 0.12 | 0.34 ± 0.11 | 0.18 ± 0.05 | 1.43 ± 0.24 | 1.09 ± 0.15 |
|  | Left | 1.07 ± 0.18 | 1.34 ± 0.22 | 0.03 ± 0.01 | 0.13 ± 0.10 | 0.38  ± 0.12 | 0.32 ± 0.12 | 0.14 ± 0.03 | 0.45 ± 0.15 | 0.88 ± 0.04 | 1.67 ± 0.38 |
