# Supplementary material for: Social dominance in rats: effects on cocaine self-administration, novelty reactivity and dopamine receptor binding and content in the striatum
Source: Psychopharmacology (Berl). 2015 Nov 10;233:579–89. doi: 10.1007/s00213-015-4122-8 (PMC4726718; doi:10.1007/s00213-015-4122-8)
Supplement: Supplementary file 3 — (DOC 35 kb) [file 213_2015_4122_MOESM3_ESM.doc]

**Supplementary Table 3:** Post-mortem levels of NA, DA, DOPAC, 5-HT and 5-HIIA within striatal sub-regions of dominant (dom) and subordinate (sub) rats. Data are expressed as pmol/mg of tissue. DLS, dorsolateral striatum; NAc, nucleus accumbens. Significant differences are shown in bold.

| Region | Side | NA | | DA | | DOPAC | | 5-HT | | 5-HIAA | |
| --- | --- | --- | --- | --- | --- | --- | --- | --- | --- | --- | --- |
|  |  | Dom | Sub | Dom | Sub | Dom | Sub | Dom | Sub | Dom | Sub |
| DLS | Right | 0.02 ± 0.01 | 0.03 ± 0.01 | 22.45 ± 8.99 | 17.10 ± 9.10 | 97.54 ± 22.61 | 79.64 ± 4.66 | 0.12 ± 0.03 | 0.10± 0.04 | 0.91 ± 0.15 | 0.73 ± 0.11 |
|  | Left | 0.03 ± 0.01 | 0.06 ± 0.03 | 23.16 ± 8.13 | 19.29 ± 8.74 | 62.10 ± 11.35 | 73.28 ± 11.24 | 0.09 ± 0.04 | 0.15± 0.08 | 0.66 ± 0.12 | 0.78 ± 0.16 |
| NAc Core | Right | 0.32 ± 0.07 | 0.34 ± 0.08 | 4.16 ± 1.77 | 9.01 ± 3.59 | 53.89 ± 10.77 | 88.24 ± 24.09 | 0.12 ± 0.04 | 0.36± 0.19 | 1.89 ± 0.28 | 2.37 ± 0.78 |
|  | Left | 0.58 ± 0.11 | 0.90 ± 0.35 | 5.26 ± 1.80 | 8.09 ± 1.19 | 72.21 ± 14.74 | 64.35 ± 4.96 | 0.16 ± 0.03 | 0.28± 0.05 | 2.00 ± 0.34 | 2.04 ± 0.38 |
| NAc Shell | Right | 1.09 ± 0.38 | 0.94 ± 0.39 | **2.20 ± 0.79** | **6.82 ± 1.19** | 48.14 ± 6.25 | 53.65 ± 7.36 | 0.31 ± 0.12 | 0.48± 0.09 | 1.75 ± 0.28 | 2.11 ± 0.15 |
|  | Left | 1.78 ± 0.48 | 1.34 ± 0.59 | **3.26 ± 1.59** | **4.47 ± 1.12** | 63.69 ± 23.76 | 50.89 ± 10.30 | 0.26 ± 0.10 | 0.46± 0.09 | 2.53 ± 0.84 | 2.61 ± 0.55 |
